# Book Reviews in Brief

**Published:** 1912-01-13

**Authors:** 


### Book Reviews in Brief.

Dental Anæsthetics: A Text-book for Students and Practitioners. By Wilfrid E. Alderson, M.D., M.S., D.P.H. (Bristol: John Wright and Sons, Ltd. Pp. 100. Price 3s. net.)

Dr. Alderson has published a most useful little manual on Dental Anæsthetics. It is in the main a *résumé* of his lectures to the students of the Newcastle-upon-Tyne Dental Hospital, and he is to be congratulated on the concise result. To practitioners who are in the habit of administering anæsthetics for dentists, and to dentists themselves, this book can be recommended with confidence. We know of no better brief exposition of the subject. The description of local analgesia has been most suitably treated by Mr. John Bolam, L.D.S., and this chapter forms a most valuable portion of the book. We may remark that the whole is carefully written, and that due attention is paid to the choice of anæsthetic and to the contraindications. This handy and economical work will, we feel sure, be much appreciated by the medical and dental professions, and it deserves to be widely known.

Two charmingly written booklets have been issued by the Rev. Henry Lansdell, D.D., the chaplain to Morden College, Blackheath. They are the outcome of a paper read before the London and Middlesex Archæological Society, and deal with "Princess Ælfrida's Charity," meaning thereby the story of the first five hundred years of the reputed Manor of Old Court, Greenwich, which is now the principal possession of the properties bequeathed to the college. This account of the author's historical researches is well worth reading.

The Treatment of Fractures by Mobilisation and Massage. By James B. Mennell, M.B., B.C. (London: Macmillan and Co. 1911. Price 12s. net.)

The method of treating fractures by mobilisation and massage is by no means a new one. It is a method more especially associated with the name of Dr. Lucas-Championniere (who supplies an introduction to this book), and is explained in every text-book, so that most students and practitioners are familiar with it. Dr. Mennell has written a very full compilation and given illustrative cases which explain the practical ways in which the method is applied. His book, however, contains little that is novel, and those who already possess the French and German manuals, or have access to the more recent works on fractures in English, will find this volume of little interest. To those who are unfamiliar with the method, Dr. Mennell's book should appeal, since it is a fair and accurate summary.

